# Reduced Mature MicroRNA Levels in Association with Dicer Loss in Human Temporal Lobe Epilepsy with Hippocampal Sclerosis

**DOI:** 10.1371/journal.pone.0035921

**Published:** 2012-05-15

**Authors:** Ross C. McKiernan, Eva M. Jimenez-Mateos, Isabella Bray, Tobias Engel, Gary P. Brennan, Takanori Sano, Zuzanna Michalak, Catherine Moran, Norman Delanty, Michael Farrell, Donncha O’Brien, Robert Meller, Roger P. Simon, Raymond L. Stallings, David C. Henshall

**Affiliations:** 1 Department of Physiology and Medical Physics, Royal College of Surgeons in Ireland, Dublin, Ireland; 2 Molecular and Cellular Therapeutics, Royal College of Surgeons in Ireland, Dublin, Ireland; 3 Department of Neurology, Beaumont Hospital, Beaumont, Dublin, Ireland; 4 Department of Pathology, Beaumont Hospital, Beaumont, Dublin, Ireland; 5 Department of Neurological Surgery, Beaumont Hospital, Beaumont, Dublin, Ireland; 6 Neuroscience Institute, Morehouse School of Medicine, Atlanta, Georgia, United States of America; 7 National Children’s Research Centre, Our Lady’s Children’s Hospital, Dublin, Ireland; Johns Hopkins University, United States of America

## Abstract

Hippocampal sclerosis (HS) is a common pathological finding in patients with temporal lobe epilepsy (TLE) and is associated with altered expression of genes controlling neuronal excitability, glial function, neuroinflammation and cell death. MicroRNAs (miRNAs), a class of small non-coding RNAs, function as post-transcriptional regulators of gene expression and are critical for normal brain development and function. Production of mature miRNAs requires Dicer, an RNAase III, loss of which has been shown to cause neuronal and glial dysfunction, seizures, and neurodegeneration. Here we investigated miRNA biogenesis in hippocampal and neocortical resection specimens from pharmacoresistant TLE patients and autopsy controls. Western blot analysis revealed protein levels of Dicer were significantly lower in certain TLE patients with HS. Dicer levels were also reduced in the hippocampus of mice subject to experimentally-induced epilepsy. To determine if Dicer loss was associated with altered miRNA processing, we profiled levels of 380 mature miRNAs in control and TLE-HS samples. Expression of nearly 200 miRNAs was detected in control human hippocampus. In TLE-HS samples there was a large-scale reduction of miRNA expression, with 51% expressed at lower levels and a further 24% not detectable. Primary transcript (pri-miRNAs) expression levels for several tested miRNAs were not different between control and TLE-HS samples. These findings suggest loss of Dicer and failure of mature miRNA expression may be a feature of the pathophysiology of HS in patients with TLE.

## Introduction

MicroRNAs (miRNAs) are a class of small (∼22 nucleotide) non-coding RNA which function as post-transcriptional regulators of gene expression by targeting protein-coding mRNAs [1]. In humans, mature miRNAs are transcribed by RNA polymerase II/III to form a primary transcript (pri-miRNA) which is then processed by the RNaseIII Drosha to pre-miRNA [2], [3]. After export to the cytoplasm, mature miRNA is produced by the action of Dicer, another RNaseIII, which generates a double-stranded miRNA from which one strand is forwarded to the RNA-induced silencing complex [3], [4]. Here, Argonaute (AGO) proteins including AGO2 perform miRNA-mediated translational repression via mRNA destabilization and degradation [3], [4].

miRNAs are abundantly expressed in the human brain and are essential for normal brain development and function [5]. Experimental deletion of Dicer from neurons results in spine loss, apoptosis and functional deficits [6], [7], [8]. Dicer deletion from astrocytes triggers seizures as well as a degenerative phenotype [9]. Accordingly, altered miRNA expression may contribute to CNS pathologies and loss of Dicer and specific miRNAs, including miR-133b and miR-9, has been reported in neurodegenerative diseases [10], [11], [12].

Temporal lobe epilepsy (TLE) is a common, chronic neurologic disorder characterized by recurrent spontaneous seizures which originate in brain structures such as the hippocampus [13]. Hippocampal sclerosis (HS) is often present in patients with refractory TLE, comprising neuron loss and gliosis within the CA1, CA3, hilus/CA4, often accompanied by dispersion of the granule cell layer [14], [15]. Loss of neurons is less common in the neocortex of TLE patients [16], [17]. Dysregulation of genes affecting neurotransmission, gliosis, neuroinflammation and apoptosis has been proposed to underlie the pathogenesis of TLE with HS [18], [19], [20] but our understanding of the mechanisms remains incomplete.

Given the phenotypes resulting from loss of Dicer and/or miRNAs from brain, we hypothesized that HS in patients with TLE may be associated with alterations in miRNA processing. We report Dicer levels are reduced in human TLE-HS tissue and this is associated with large-scale reductions in levels of mature miRNAs. These findings indicate a potential failing of mature miRNA production in the sclerotic hippocampus of TLE patients.

## Results

### Reduced Dicer Levels in Human TLE with HS

**Figure 1:**
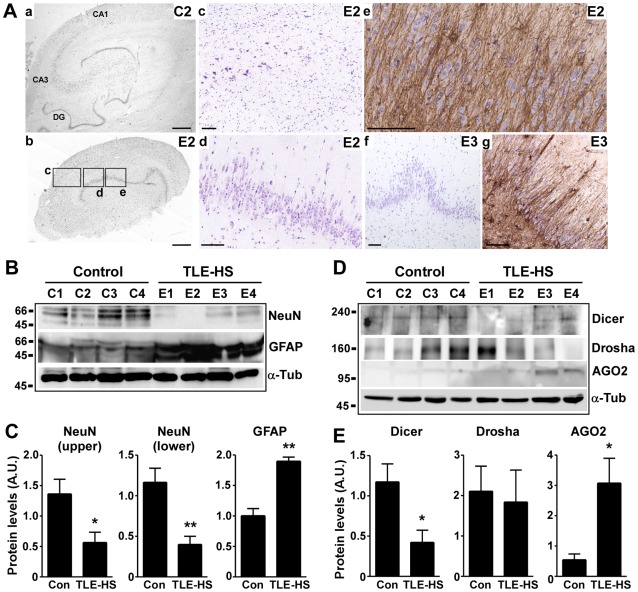
Reduced Dicer levels in TLE patients with hippocampal sclerosis. (A) Representative histology for autopsy controls and patient samples. (a) Photomicrograph showing field view of a cresyl violet-stained section from an autopsy control hippocampus (C2). (b) Photomicrograph showing a field view of the hippocampus from a TLE-HS patient (E2). Boxes in *b* indicate regions of the section from which higher-power images and other stains in *c–e* are presented. (c) Evident pyramidal neuron loss in the CA1 sector (20× lens). (d) Granule cell layer dispersion in E2. (e) GFAP immunohistochemistry showing astrogliosis within the granule cell layer. (f, g) Cresyl violet and GFAP-stained sections from patient E3 showing granule cell layer dispersion and astrogliosis (20× lens). Scale bar in *a, b*, 1 mm; *c–g*, 100 µm. (B) Representative Western blots showing protein levels of neuron (NeuN) and astrocyte (GFAP) markers in hippocampus from controls (C1–4) and TLE-HS patients (E1–4). (C) Semi-quantification of protein levels (*n* = 4 per group). Levels of the uppermost band (∼66 kD) and a lower band (48 kD) were lower in TLE-HS samples, whereas GFAP (uppermost band analyzed) was higher. (D, E) Representative Western blots and graphs showing protein levels and semi-quantification for Dicer, Drosha and AGO2. *p<0.05; **p<0.01 compared to controls. Protein levels corrected to α-Tubulin (α-Tub).

Human autopsy control and TLE patient clinical data are reported in Tables 1 and 2. The average age of TLE patients was 37.2 years which was not different from autopsy controls (31.6 years; P = 0.236) and each group included males and females. Nissl-stained sections from autopsy controls displayed typical hippocampal architecture (Fig. 1A). In contrast, HS samples from TLE patients displayed extensive pyramidal neuron loss and gliosis, with some also showing granule neuron dispersal (Figure 1A, Table 2).

**Table 1:** Autopsy control details.

Identifier	Gender	Age, y	Tissue	Cause of death	PMI, h
C1	M	42	Hippocampus	Multiple injuries, road traffic accident	8
C2	M	37	Hippocampus	Arteriosclerotic cardiovascular disease, ruptured aortic aneurysm	12
C3	M	28	Hippocampus and T.L. neocortex	Multiple injuries, road traffic accident	7
C4	F	26	Hippocampus and T.L. neocortex	Cardiac tamponade	12
C5	F	25	T.L. neocortex	Multiple injuries, road traffic accident	7

Key: F, female; h, hours; M, male; PMI, post-mortem interval; T.L., temporal lobe; y, years.

**Table 2:** TLE patient clinical and pathological details.

Identifier	Gender	Age, y	Tissue	Diagnosis and other pathology findings
E1	M	37	Hippocampus	Hippocampal sclerosis; Specimen comprises dentate fascia and pyramidal cell layer. Loss of cells in dentate fascia, and considerable loss of pyramidal cells with accompanying gliosis
E2	M	23	Hippocampus	Hippocampal sclerosis; Specimen comprises pyramidal layer and dentate fascia. Minimal GCD, severe pyramidal neuron loss in CA2 and CA3 with accompanying astrogliosis
E3	M	39	Hippocampus	Hippocampal sclerosis; Specimen contains hippocampus, dentate fascia with dispersal of neurons, but pyramidal layer not included
E4	F	31	Hippocampus	Hippocampal sclerosis (mild); Specimen comprises dentate fascia, CA4 and CA3 regions showing moderate gliosis (GFAP histochemistry)
E5	F	34	Hippocampus	Hippocampal gliosis; Specimen comprises hippocampus including dentate fascia and CA4 and showing moderate gliosis (GFAP histochemistry). No evidence of tumor, infection or vascular malformation
E6	F	43	Hippocampus	Hippocampal gliosis; Specimen comprises dentate fascia and CA4 region. Focal gliosis in CA4 region
E7	M	29	Hippocampus	Specimen comprises dentate fascia and pyramidal cell layer. Astrocytic hyperplasia with pyramidal cell layer with neuron loss
E8	M	48	Hippocampus	Hippocampal gliosis; Specimen comprises dentate fascia and hippocampal pyramidal cell layer. Very minor degree of astrocytic hyperplasia (white matter). Pyramidal layer reasonably well populated with neurons, no evidence of any dispersal of neurons in the dentate fascia
E9	M	27	T.L. neocortex	Normal cortical laminar pattern, cyto-architecture well maintained, no evidence of any inflammatory or neoplastic process and no evidence of gliosis or other epilepsy-associated lesion
E10	M	53	T.L. neocortex	Unremarkable although changes to subcortical white matter include foci of hypercellularity (astrocytes and oligodendroglia and endothelial hyperplasia. Some dysplastic neurons
E11	M	45	T.L. neocortex	Normal. Hexilaminar pattern, normal subcortical white matter, no cortical dysplasia or neuronal ectopia, no inflammatory process or abnormal vessels

*Key;* F, female; GCD, granule cell dispersion; M, male; T.L. temporal lobe; y, years.

Hippocampal samples from TLE patients were divided into two sets for further analysis (TLE1–4 and TLE5–8), with the samples displaying the most severe HS being in the first group (see Table 2, Figure 1B). Confirming pathological observations, Western blot analysis showed levels of the mature neuron marker NeuN were significantly lower in HS samples from TLE patients 1–4 compared to controls (Figure 1B, C). Protein levels of glial fibrillary acidic protein (GFAP), an astrocyte marker, were significantly higher in these TLE-HS samples compared to controls (Figure 1B, C).

Next, we examined hippocampal levels of key enzymes in the miRNA biogenesis pathway. Dicer was detected at its predicted weight of ∼220 kD in all control samples (Figure 1D). In contrast, Dicer levels were significantly lower in TLE-HS samples (Figure 1D, E). We also detected higher levels of a lower-weight Dicer species, presumably cleaved Dicer in TLE-HS samples (Figure S1A).

Drosha, which processes pri-miRNA into pre-miRNA [2], was detected at ∼160 kD in control and HS samples from TLE patients. There was no significant difference in protein levels between the groups (Figure 1D, E). AGO2 was detected in control hippocampus and protein levels were significantly higher in HS samples from patients with TLE (Figure 1D, E).

### Reduced Mature miRNA Expression in Human TLE with HS

**Figure 2:**
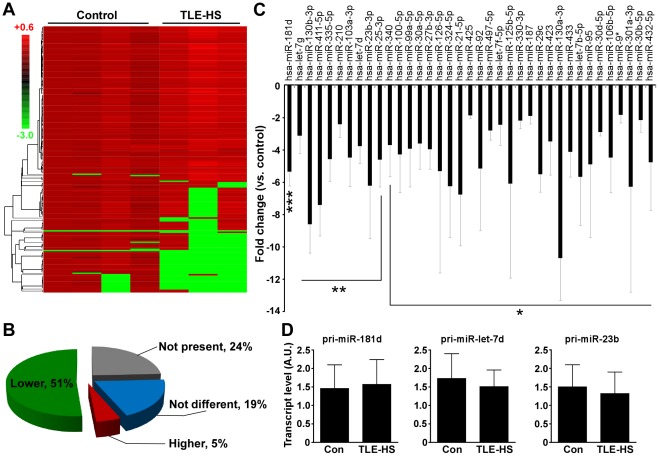
Reduced mature miRNA expression in human TLE-HS. (A) Heat-map depiction of raw miRNA expression data for 188 of the expressed miRNAs from controls (C1–C4) and TLE-HS samples (TLE1, TLE2 and TLE4). Note the reduction in miRNA levels (green bars) in TLE-HS samples. Scale bar to left indicates fold differences. (B) Pie chart summarizing the findings from profiling miRNA levels in human TLE-HS samples. Over half the expressed miRNAs were found to be lower than in controls. (C) Graph showing the expression of the significantly down-regulated miRNAs in TLE-HS samples. Individual Ct values from each TLE sample were normalized to the average of the Ct values from the control group. The graph represents the average of the 2^−ΔΔCt^ of each TLE sample. *p<0.05; **p<0.01; ***p<0.001 compared to control (from *n* = 4 controls versus *n* = 3 TLE-HS samples). Note, Hsa is nomenclature denoting human miRNA sequence. (D) Graphs (*n* = 3–4 per group) showing expression (2^−ΔΔCt^, normalized to *Gapdh*) for a selection of pri-miRNAs in the same control and TLE-HS samples.

Since Dicer is a critical enzyme in the production of mature miRNAs we hypothesized that mature miRNA levels may be reduced in TLE-HS samples. To test this, we profiled mature miRNA levels in hippocampus from controls (C1–4) and the samples from TLE patients displaying reduced Dicer (TLE 1, 2 and 4; sample 3 was omitted due to insufficient material; see Methods for details). Using Taqman low density arrays (TLDA) [21] we screened 380 human miRNAs of which 198 miRNAs (52.1%) were called present in control human hippocampus (Figure 2A and data not shown). The 40 most abundantly expressed miRNAs in human autopsy control hippocampus, based on Ct values, are presented in Table S1. The expression of miRNAs was substantially different in HS tissue from TLE patients (Figure 2A–C). Only 150 miRNAs were called present in TLE-HS samples, with 48 (24.2%) of the control-expressed miRNAs not detected. No miRNAs were detected in TLE-HS samples but not controls. Of the expressed miRNAs, 39 (19.7% of those present in controls) showed no difference in expression from control (<1.5 fold difference), while 101 (51.0% of the number in controls or 67.3% of those expressed in TLE-HS) were present at lower levels than in controls (Figure 2A, B, C). Among the down-regulated miRNAs, 37 of the 150 were significantly different from controls (Figure 2C). Only 10 miRNAs were expressed at higher levels in TLE samples than in controls (Figure 2B and Figure S2).

### Pri-miRNA Expression is not Reduced in Human TLE-HS

We next measured pri-miRNA transcript levels for a selection of miRNAs which displayed significantly reduced mature levels in TLE-HS (Figure 2C, D). Measurement of pri-miRNA transcript levels for four pri-miRNAs (∼10% of those significantly reduced in the mature miRNA screen) revealed no significant differences between TLE-HS samples and controls for pri-miR-181d, pri-miR-130a, pri-miR-let-7d and pri-miR-23b (Figure 2D and data not shown).

As a control to ensure mature miRNA differences were not the result of an autopsy-related change in miRNA levels, we simulated a post-mortem interval using mouse brain (Figure S3). Levels of three mature miRNAs tested did not change with an autopsy delay of up to 8 h (Figure S3A). Levels of Dicer protein were reduced as a function of simulated autopsy delay whereas levels of Ago2 protein increased with simulated autopsy delay time (Figure S3B). Simulated autopsy delay had no effect on protein levels of Drosha (Figure S3B).

### Bioinformatics Analysis of Pathways Impacted by Reduced Mature miRNA Expression in TLE-HS

To gain insight into how the suppressed miRNA profile in TLE-HS tissue might affect signalling pathways we identified potential mRNA targets of the significantly down-regulated miRNAs (see Methods, Figure S4). For cellular component, genes associated with the nucleus were predicted to be most impacted. Among biological processes and molecular function, the pathways predicted to be affected included nucleic acid metabolism and transcriptional regulation (Figure S4).

### Normal Dicer Levels in Human TLE Neocortex

**Figure 3:**
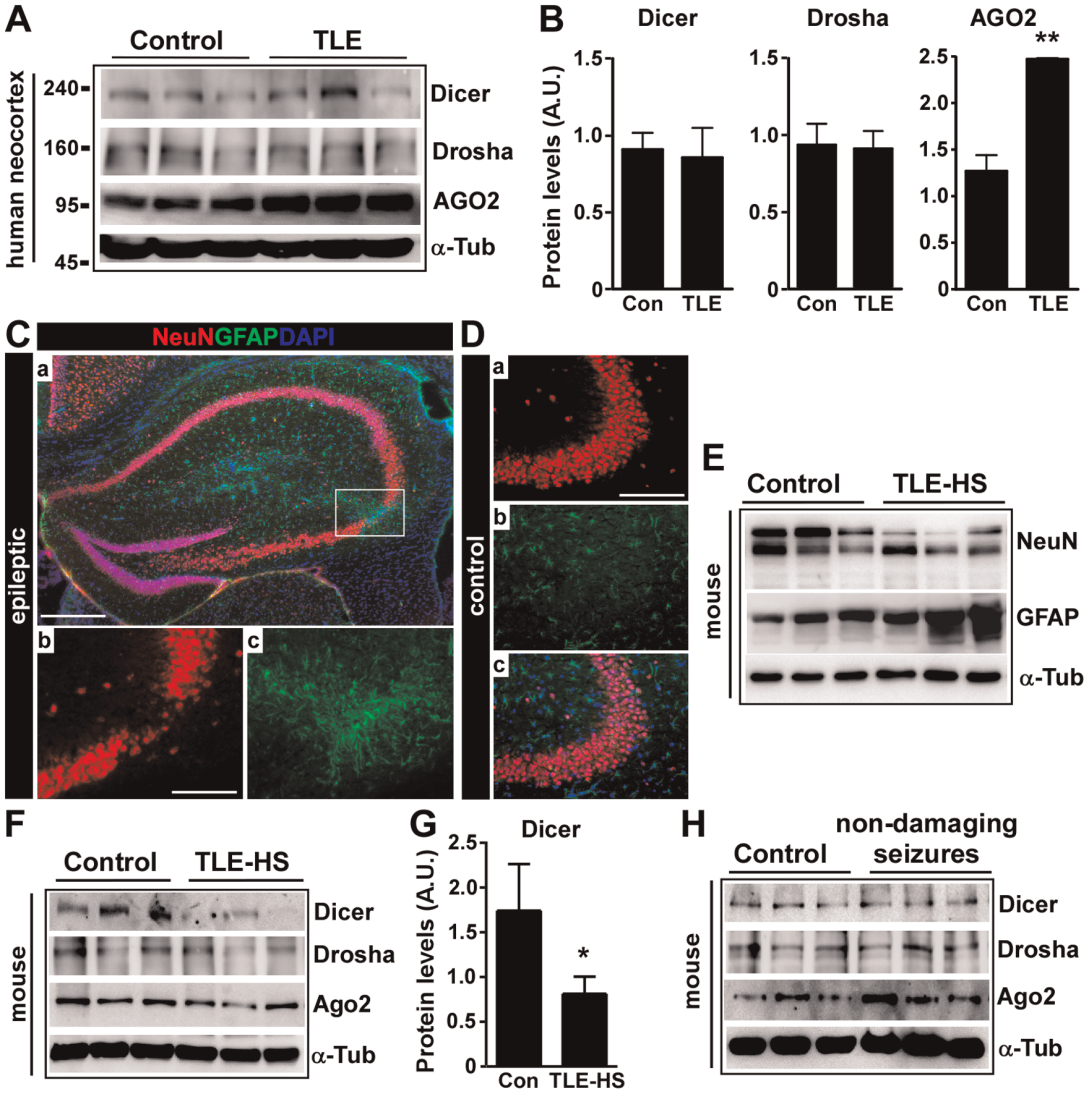
miRNA biogenesis components in neocortex and in experimental epilepsy. (A) Representative Western blots showing expression of Dicer, Drosha and AGO2 in neocortex samples from TLE patients (TLE 9–11) and controls (C3–C5). (B) Graphs (*n* = 3 per group) showing semi-quantification of levels of each protein. **p<0.05 compared to controls. Protein levels corrected to α-Tubulin (α-Tub). (C) Representative immunohistochemistry illustrating the hippocampal damage in epileptic mice. (a) Field view (4× lens) showing triple-stained section from an epileptic mouse 21 days after status epilepticus. Box highlights main lesion area in CA3. *b,c* are 20× lens field views of the lesioned CA3 area showing reduced NeuN (red) and increased GFAP (green) staining. (D) Representative views of the CA3 from a control mouse at 21 days which did not undergo status epilepticus. *a–c* show NeuN (red), GFAP (green) and a merge of these with DAPI overlay. Note the intact neuron population and minimal GFAP staining. Scale bar in C*a*, 500 µm; C*b*, and D*a*, 100 µm. (E, F) Representative Western blots (*n* = 1 per lane) showing protein levels of NeuN, GFAP, Dicer, Drosha and Ago2 in CA3 samples from control and epileptic (21 day) mice (TLE-HS). (G) Graph quantifying reduction in hippocampal CA3 Dicer levels in epileptic animals (*n* = 7–8 per group; *p<0.05 compared to control). Protein levels corrected to α-Tub. (H) Representative Western blots (*n* = 1 per lane) show non-damaging seizures do not cause a reduction of Dicer levels. α-Tubulin is included as a guide to protein loading.

Levels of Dicer, Drosha and AGO2 in the second set of TLE samples in which hippocampal pathology was moderate/minimal (see Table 2) were not significantly different from controls (data not shown). Dicer and Drosha levels in human neocortex were also not significantly different between TLE patients and controls (Figure 3A, B). AGO2 levels were significantly higher in TLE neocortex samples compared to controls (Figure 3A, B).

### Loss of Hippocampal Dicer in Experimental TLE

To support the patient findings, we measured Dicer levels in the sclerotic hippocampus of epileptic mice. Epilepsy was induced by stereotaxic microinjection of kainic acid into the amygdala to trigger status epilepticus. This results in the emergence of recurrent spontaneous seizures in all animals within the first week and continuing thereafter [22]. Consistent with previous reports [22], [23], tissue sections from mice 21 days after status epilepticus displayed neuron loss and astrogliosis within the CA3 subfield (Figure 3C, D). This was supported by Western blot analysis of NeuN and GFAP levels (Figure 3E).

Dicer levels were significantly lower in hippocampal CA3 samples from epileptic mice (Figure 3F, G). In addition, there were higher levels of cleaved Dicer (Figure S1B). Hippocampal levels of Drosha and Ago2 were not different between controls and epileptic mice (Figure 3F and data not shown). Dicer levels were not changed in the hippocampus by brief, non-damaging seizures induced in mice by intraperitoneal injection of a low-dose of kainic acid (Figure 3H).

## Discussion

Dicer is essential for production of most mature miRNAs and its loss from neurons or astrocytes results in miRNA down-regulation, neuronal dysfunction and apoptosis, seizures and cognitive deficits [6], [7], [9]. Thus, the phenotype of Dicer deletion shares important commonalities with the hallmarks of TLE and its underlying pathophysiology [13], [15], [18]. Altered miRNA expression has been suggested to be a causal factor in several CNS diseases [10], [11], [12], [24] but the present study is the first to both investigate miRNA biogenesis and profile miRNA expression in human TLE. We found a selective reduction in Dicer levels in tissue from TLE patients with severe HS. We also found Dicer levels were reduced in experimental TLE-HS. Since Dicer levels were normal in less sclerotic human TLE tissue, and are not changed after status epilepticus [21] or brief non-harmful seizures (Figure 3H), the loss of Dicer appears specific to TLE with HS. These findings represent a significant advance on previous work that showed dysregulation of miRNAs after seizures and in epilepsy [21], [25], [26], [27]. Given the pathological consequences of Dicer loss in brain [6], [7], [9], [10] we speculate that loss of Dicer may contribute to the pathogenesis of TLE-HS. Hippocampal sclerosis therefore appears to share loss of Dicer in common with certain neurodegenerative diseases [28], [29].

We do not know the cause of Dicer loss but since Dicer is expressed in neurons [30] the finding may simply reflect severe neuron loss in the tissue. Other biogenesis components, however, did not show reduced levels and astrocytes, which also express Dicer [9], were increased in HS tissue. Indeed, the selective deletion of Dicer from neurons only reduces hippocampal protein levels by ∼60%, presumably due to residual glial Dicer [8]. Taken together, this favors a more specific cause of the down-regulation. In addition to possible transcriptional downregulation, Dicer is known to be a substrate for cleavage-dependent inactivation by caspases [31] and caspases are activated in HS tissue from TLE patients [20]. Caspase-mediated Dicer cleavage can also convert Dicer into a pro-apoptotic DNase [32] and cells positive for DNA fragmentation are present in resected TLE material [33], [34]. We detected possible Dicer cleavage in both human and experimental epilepsy, although the lower-weight species was most similar to a reported calpain-generated fragment [30]. Identifying ways to rescue Dicer or block its down-regulation could offer a means to reconstitute miRNA expression in HS tissue.

AGO2 levels were higher in human TLE-HS and neocortex, suggesting epileptic seizures may up-regulate AGO2. The temporal cortex samples from TLE patients displayed normal levels of both Dicer and Drosha. It is notable that AGO2 is capable of processing certain miRNAs without Dicer, including miR-451 [35], [36], and miR-451 was among the few upregulated miRNAs in TLE-HS tissue. Thus, elevated AGO2 in human TLE may contribute to maintaining levels of this miRNA.

Recent work suggests there are about 600 miRNAs expressed in the human brain, although a collection of ∼20 miRNAs account for nearly 90% [37]. The present study included profiling of mature miRNA expression in normal human autopsy hippocampus, and nearly 200 were detected. Among those expressed, 15 have previously been reported as abundant in human dorsolateral prefrontal cortex [37], including miR-26a and miR-125b. Among other abundant miRNAs were known brain-enriched miRNAs, including miR-9 [38], and miR-132 [8] and astrocyte-expressed miRNAs such as miR-29a [39].

The second major finding in the present study was that TLE-HS tissue displayed lower expression of many mature miRNAs. Two-thirds of the detected miRNAs were present at lower levels than in controls, and nearly a quarter of the control-expressed miRNAs were not detected in TLE-HS samples. This indicates a failure or collapse of mature miRNA expression in human TLE-HS. Our data are in congruence, therefore, with the effect of experimental inactivation of Dicer which results in reduced levels of many, but not all, miRNAs [6], [7], [40], [41]. The similar level of several pri-miRNAs and Drosha in TLE-HS is also supportive of the defect in mature miRNA production being at the level of Dicer. Notably, miR-486 levels do not change in the hippocampus after Dicer loss [8] and this miRNA was not differently expressed in TLE-HS in our study. The persistence of some mature miRNA production in the sclerotic hippocampus may be due to residual Dicer activity, longevity of the mature miRNAs in hippocampus after Dicer loss [7], [8], or compensation by other proteins within the miRNA biogenesis pathway [35], [36]. Although loss of Dicer is the most obvious explanation for the observed large-scale reduction in miRNA levels, differences in expression of other miRNA biogenesis components such as the Drosha partner DiGerorge syndrome chromosome 8 (DGCR8) or Dicer partner transactivating response RNA-binding protein (TRBP), would also be likely to affect mature miRNA levels [40], [42], [43], [44], and transcription factors which regulate miRNA levels continue to be identified [45].

How might loss of miRNA expression in TLE-HS tissue contribute to disease pathogenesis? Broadly, a collapse of the miRNA system would lead to the loss of a major mechanism regulating expression of protein-coding genes which, as our bioinformatics analysis supports, could impact diverse cellular processes. More specifically, Dicer loss from astrocytes provokes a reactive astrocytosis, which is known to contribute to seizure generation [9], [46]. Loss of miRNA biogenesis also produces degenerative changes to dendritic morphology that have been observed in experimental and human epilepsy [7]. A failure of mature miRNA production due to Dicer deletion causes neuronal death by apoptosis [7], [9] and previous human data show higher protein levels in TLE-HS for genes implicated in apoptosis, including caspases [47]. Loss of certain miRNAs has been implicated in neurodegenerative diseases, including miR-9* [48] and miR-106b [49]. Both these miRNAs were present at lower levels in TLE-HS samples in our study. Thus, reduced miRNA levels may play a role in cell loss in TLE-HS, although whether neuron loss is a causal event in epileptogenesis remains debated [50], [51].

There are a number of potential caveats to consider in the present study. Larger cohorts of patients, stratified for pathology and/or seizure type and analysis of non-TLE epilepsy material will be needed to corroborate our findings. Certain miRNAs were not detected that might have been expected, for example miR-146a which was reported to be over-expressed in TLE [25]. Another potential confounder is anti-epileptic drug exposure. Our simulated post-mortem interval experiments exclude any key finding being an artefact of autopsy delay. Indeed, autopsy delay would tend to underestimate the difference between control and TLE samples. Finally, the use of the TLDA platform to quantify miRNAs lacks the extensive coverage and quantitation possible with deep sequencing, including the ability to detect variants of mature miRNAs, such as isomiRs and mirtrons [52], [53].

In summary, the present study provides evidence of dysfunction of miRNA biogenesis in HS tissue from TLE patients. Future efforts might be directed to determining whether restitution of Dicer to such tissue restores mature miRNA production and influences the epileptic phenotype.

## Methods

### Human Brain Tissue Samples

This study was approved by the Ethics (Medical Research) Committee of Beaumont Hospital, Dublin (ERC/IRB 05/18) and written informed consent was obtained from all patients. Control (autopsy) hippocampus (*n* = 4) and temporal cortex (*n* = 3) was obtained from five individuals from the NICHD Brain and Tissue Bank for Developmental Disorders at the University of Maryland, Baltimore, MD. Fresh frozen specimens were from individuals without known neurological disease (Table 1). Patients (*n* = 11) were referred for surgical resection of the temporal lobe by an epileptologist (N.D.) following neurological assessment, video-EEG recording and MRI/neuroimaging. Each patient was determined to have medically intractable TLE with an history of recurring seizures (Table 2). All patients were taking anticonvulsant medication prior to surgery. Patients underwent left or right temporal lobe resection and the hippocampus and/or adjacent neocortex was obtained. A portion of each specimen was retained for pathology (see Table 2) with the remainder frozen in liquid nitrogen and stored at –70°C until use. A pathologist (M.F.) assessed hippocampus (*n* = 8) and neocortex (*n* = 3) as part of the pathologic evaluation, addressed the degree of hippocampal sclerosis, and described other pathologic changes. Each sample was divided approximately equally and processed either for miRNA extraction or protein analysis.

### Animal Model of TLE

Animal experiments were performed in accordance with the principles of the European Communities Council Directive (86/609/EEC) and were approved by the Research Ethics Committee of the Royal College of Surgeons in Ireland (REC 205). Procedures were undertaken as previously described with modifications [21], [22]. Adult male C57BL/6 mice (20–25 g) were obtained from Harlan, UK. Briefly, mice were equipped with skull-mounted EEG electrodes and a guide cannula under surgical anaesthesia and then allowed to recover before triggering status epilepticus by microinjection of kainic acid (Sigma-Aldrich, Dublin, Ireland) into the basolateral amygdala nucleus (0.3 µg in 0.2 µl phosphate-buffered saline). Non-seizure control mice received intraamygdala vehicle. After 40 min, mice were administered lorazepam (6 mg/kg, i.p.) to curtail seizures and reduce morbidity and mortality. Animals were euthanized 21 days later and saline-perfused to remove intravascular blood components. The CA3 subfield was extracted and processed for protein analysis, as described [21]. To model brief non-harmful seizures, mice received a single intra-peritoneal (i.p.) injection of kainic acid (15 mg/kg). This causes mild behavioral changes, including freezing and twitching but does not cause status epilepticus and does not result in any neuronal death [21]. Animals were killed 4 h later and brains processed as below.

### miRNA Extraction and Expression Profiling

Total RNA was extracted from tissue samples using the miRNeasy kit (Qiagen, West Sussex, UK) as per manufacturer’s instructions to obtain an enrichment of small RNAs. Quality and quantity of RNA was measured using a Nanodrop Spectrophotometer (Thermoscientific, Loughborough, UK) and RNA dilutions made up in nuclease-free water. Reverse transcription of 100 ng of miRNA was carried out using stem-loop Multiplex primer pools (Applied Biosystems, Paisley, U.K.). The miRNA screen was carried out on the 7900HT Fast Realtime System using TaqMan Low-Density Arrays (TLDA) (TaqMan TLDA MicroRNA Assays v1.0 containing 380 human microRNAs assays; Applied Biosystems). A relative fold change in expression of the target gene transcript was determined using the comparative cycle threshold method (2^−ΔΔCT^). miRNAs were called present when detected under the cut-off of 35 cycles of amplification in 3 out of 4 samples (controls) or 2 out of 3 (TLE patients). For comparative expression analysis, data for each miRNA were normalized to the average Ct value for each patient sample, as reported [54]. The Ct is the number of cycles required for the fluorescent signal to cross the threshold/background level. Thus, the lower the Ct value the greater the amount of RNA in each sample, with a difference of 0.5–1 cycle in the Ct representing ∼2 fold difference in expression of the gene. A threshold of ≥1.5 fold was considered increased or decreased compared to control, with statistical testing performed using Student’s t-test, as elsewhere [55]. To visualize the data we used Array Star software from DNASTAR (http://www.dnastar.com) with control sample C4 used as the baseline. Hierarchical clustering of the miRs was performed using an Euclidean distance metric.

For miRNA specific primers and quantitative real-time PCR [21], reverse transcription for individual qPCRs was carried out using 100 ng of total RNA and the High-Capacity Reverse Transcription Kit (Applied Biosystems). RT specific primers for mouse miRNAs mmu-miR-19a, mmu-miR-132 and mmu-miR-326 (Applied Biosystems) were used. Individual qPCRs were carried out on the 7900HT Fast Realtime System (Applied Biosystems) and RNU19 was used for normalization.

For analysis of primary transcripts (pri-miRNA), double-stranded cDNA was synthesized from 176 ng RNA remaining from samples C1–4 and TLE 1, 2 and 4, using SuperScript II Reverse Transcriptase (Biosciences, Dun Laoghaire, Ireland). Next, cDNA was amplified using the Pre-amplification Kit as per manufacturer’s guidelines (Applied Biosystems). PCR analysis was performed using cDNA in triplicate on the 7900 HT Fast Realtime system (Applied Biosystems) for the following primary-miRNA transcripts using Applied Biosystems pri-miRNA guidelines; pri-mir181d (Hs03303910_pri), pri-mir130a (Hs03303108_pri), pri-let7d (Hs03302562_pri) and pri-mir23b (Hs03303058_pri). Minus reverse-transcription and non-template controls were routinely used to rule out genomic DNA and cross-well contamination respectively. *Gapdh* (Hs99999905_m1) was used for normalization. A relative fold change in expression compared to control was performed using the comparative cycle threshold method (2^−ΔΔCt^).

### miRNA Target Prediction Bioinformatics

Predicted mRNA targets of significantly regulated miRNAs were identified using www.targetscan.org. For identification of the most impacted GO (gene ontology) processes, lists of the top 30 highest scored mRNA targets based on pairing complementarity [56] were analyzed using DAVID (http://david.abcc.ncifcrf.gov/).

### Histology

Autopsy control and patient tissue sections were fixed and stained with cresyl violet. GFAP immunohistohemistry was carried out using anti-GFAP (Dako Diagnostics Ireland Ltd, Dublin, Ireland), with diaminobenzdine enhancer on an automated system. Slides were scanned on a Leica microsystems whole slide imaging platform. For mouse studies, fresh frozen cryo-sections (12 µm) were post-fixed, blocked in goat serum and incubated with anti-NeuN (Millipore, Tullagreen, Co Cork, Ireland) and anti-GFAP antibodies, followed by secondary antibodies conjugated to AlexaFluor 488 or 568, counterstaining with 4′,6 diamidino-2-phenylindole (DAPI), as described [22]. Staining was visualized using a Nikon2000s epifluorescence microscope and images taken using a Hamamatsu Orca 285 camera.

### Western Blotting

Western blotting was performed as previously described [21]. Briefly, samples were homogenized and lysed in buffer containing a protease inhibitor cocktail. Protein (30 µg) was then separated by 8–10% sodium dodecyl sulfate-polyacrylamide gel electrophoresis, transferred to nitrocellulose membranes, and incubated with the following antibodies: AGO2 and Drosha (Cell Signaling Technology, Beverly, MA, USA), Dicer-1 (Santa Cruz Biotechnology, Santa Cruz, CA, USA), NeuN (Millipore), GFAP and α-Tubulin (Sigma-Aldrich Ireland Ltd, Arklow, Ireland). Membranes were then incubated with horseradish peroxidase-conjugated secondary antibodies (Jackson ImmunoResearch, Plymouth, PA, USA) and bands visualized using Supersignal West Pico chemiluminescence (Pierce, Rockford, IL, USA). Images were captured using a Fuji-Film LAS-300 (Fuji, Sheffield, UK) and densitometry performed using AlphaEaseFC4.0 gel-scanning integrated optical density software (Alpha Innotech, San Leandaro, CA, USA).

### Data Analysis

All data are presented as mean ± s.e.m. Comparison of group ages and protein densitometry was performed using unpaired *t*-test. Significance was accepted at *p*<0.05.

## Supporting Information

Figure S1 **Dicer immunoreactive bands at 75 kD in human and experimental TLE-HS.** Western blots showing the presence of a cleaved band of Dicer in experimental and human epilepsy (MS Word).
(DOC)

Figure S2 **Upregulated miRNAs in human TLE-HS tissue.** Graph showing the expression levels of miRNAs in human TLE-HS tissue for which expression was non-significantly higher than in controls (MS Word).
(DOC)

Figure S3 **Effects of simulated autopsy delay on hippocampal miRNA levels and miRNA biogenesis components.** Results of simulated autopsy delay experiments using mouse brain. RT-PCR analysis of three miRNAs and Western blots showing effects on protein levels of Dicer, Drosha and Ago2 (MS Word).
(DOC)

Figure S4 **Bioinformatic analysis of genes impacted by significantly down-regulated miRNAs in human TLE-HS.** Gene ontology analysis showing cellular component, biological process and molecular functions of the predicted mRNA targets of down-regulated miRNAs in human TLE-HS tissue (MS Word).
(DOC)

Table S1 Top 40 miRNAs expressed in autopsy control human hippocampus. Table listing the 40 mature miRNAs in autopsy control human hippocampus most enriched based on Ct value (MS Word).
(DOC)
