# Supplementary material for: Reduced Mature MicroRNA Levels in Association with Dicer Loss in Human Temporal Lobe Epilepsy with Hippocampal Sclerosis
Source: PLoS One. 2012 May 15;7(5):e35921. doi: 10.1371/journal.pone.0035921 (PMC3352899; doi:10.1371/journal.pone.0035921)
Supplement: Figure S1 — Dicer immunoreactive bands at 75 kD in human and experimental TLE-HS. Western blots showing the presence of a cleaved band of Dicer in experimental and human epilepsy (MS Word). (DOC) [file pone.0035921.s001.doc]

**Supplementary data Figure S1**


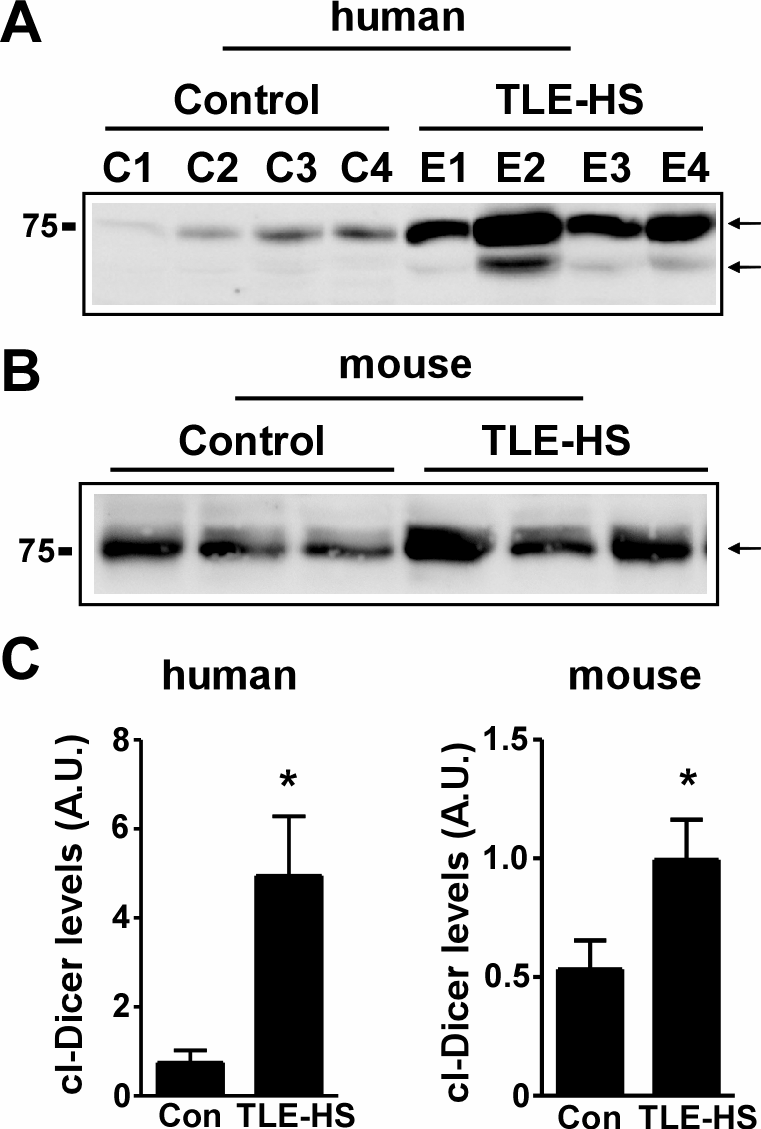


**Supplementary data Figure S1** *Dicer immunoreactive bands at 75 kD in human and experimental* *TLE-HS.* (A) Representative Western blot (*n* = 1 per lane) showing a ~75 kD band detected by immunoblotting with anti-Dicer antibody which may correspond to the reported calpain-cleaved form of Dicer. (B) Representative Western blot (*n* = 1 per lane) showing the ~75 kD band following immunoblotting of microdissected hippocampal CA3 subfield from mice three weeks after status epilepticus with anti-Dicer antibody. Arrows indicate band. For loading controls see respective panels in Figure 1D and Figure 3F. (C) Graphs showing semi-quantitative analysis of the cleaved (cl) Dicer fragment in each tissue (*p* < 0.05; *n* = 4 per group for human; *n* = 7-8 per group for mouse).
