# Supplementary material for: Reduced Mature MicroRNA Levels in Association with Dicer Loss in Human Temporal Lobe Epilepsy with Hippocampal Sclerosis
Source: PLoS One. 2012 May 15;7(5):e35921. doi: 10.1371/journal.pone.0035921 (PMC3352899; doi:10.1371/journal.pone.0035921)
Supplement: Figure S2 — Upregulated miRNAs in human TLE-HS tissue. Graph showing the expression levels of miRNAs in human TLE-HS tissue for which expression was non-significantly higher than in controls (MS Word). (DOC) [file pone.0035921.s002.doc]

**Supplementary data Figure S2**


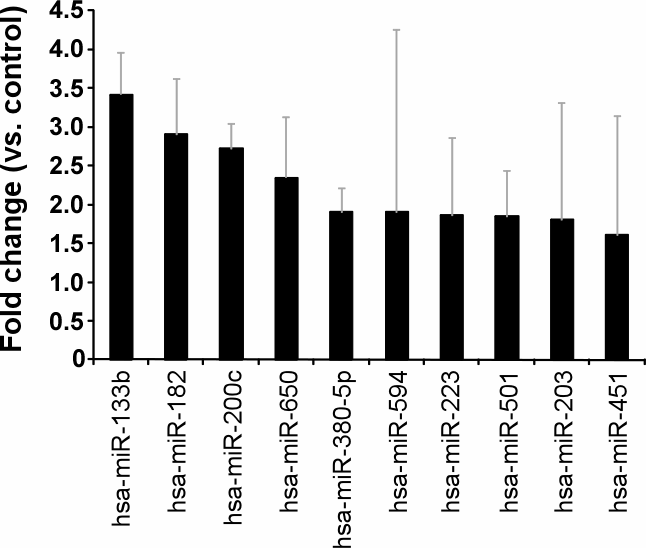


**Supplementary data Figure S2.** *Upregulated miRNAs in human TLE-HS tissue.* Graph shows miRNA levels for the small number (6.7 % of those expressed in TLE tissue) which were increased (>1.5 fold) in TLE samples compared to control. Differences were not, however, statistically significant (P > 0.05, *n* = 4 controls; *n* = 3 TLE).
