# Supplementary material for: Reduced Mature MicroRNA Levels in Association with Dicer Loss in Human Temporal Lobe Epilepsy with Hippocampal Sclerosis
Source: PLoS One. 2012 May 15;7(5):e35921. doi: 10.1371/journal.pone.0035921 (PMC3352899; doi:10.1371/journal.pone.0035921)
Supplement: Figure S3 — Effects of simulated autopsy delay on hippocampal miRNA levels and miRNA biogenesis components. Results of simulated autopsy delay experiments using mouse brain. RT-PCR analysis of three miRNAs and Western blots showing effects on protein levels of Dicer, Drosha and Ago2 (MS Word). (DOC) [file pone.0035921.s003.doc]

**Supplementary data Figure S3**


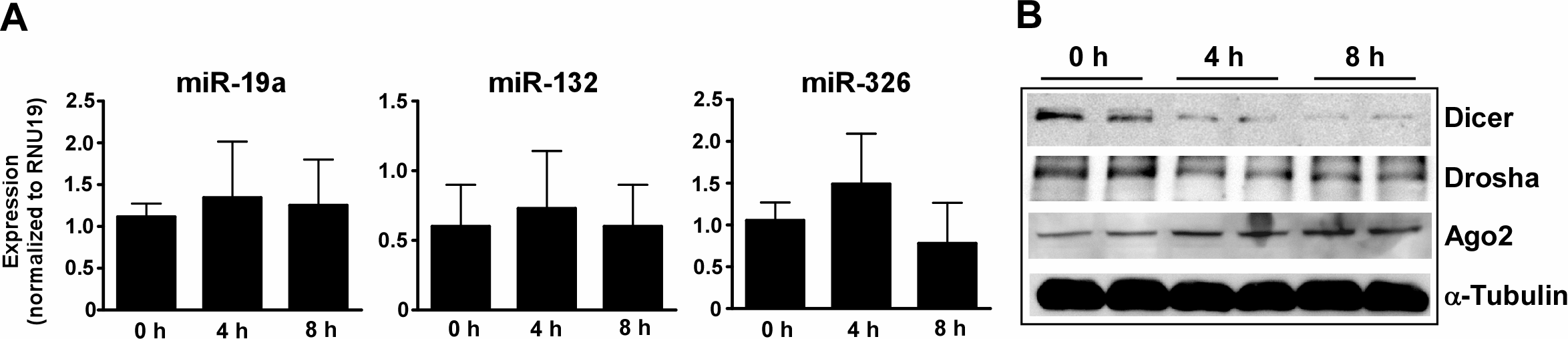


**Supplementary data Figure S3.** *Effects of simulated autopsy delay on hippocampal miRNA levels and miRNA biogenesis components.* (A) Graphs show miRNA levels (normalized to RNU19) determined by individual RT-PCR. Mice were killed and left at room temperature for 0, 4 or 8 h (*n* = 3 per group). Hippocampus was then dissected, miRNAs extracted and the three miRNAs measured. No changes for any miRNA were found. (B) Western blots for the three main biogenesis proteins studied. Note, autopsy delay results in a reduction in Dicer (*p* < 0.05 for 4 and 8 h compared to 0 h). Drosha levels underwent a small, non-significant reduction (*p* > 0.05), while levels of Ago2 increased (*p* < 0.05 for 4 h and 8 h compared to 0 h) (*n* = 4 per group).
