# Supplementary material for: Reduced Mature MicroRNA Levels in Association with Dicer Loss in Human Temporal Lobe Epilepsy with Hippocampal Sclerosis
Source: PLoS One. 2012 May 15;7(5):e35921. doi: 10.1371/journal.pone.0035921 (PMC3352899; doi:10.1371/journal.pone.0035921)
Supplement: Figure S4 — Bioinformatic analysis of genes impacted by significantly down-regulated miRNAs in human TLE-HS. Gene ontology analysis showing cellular component, biological process and molecular functions of the predicted mRNA targets of down-regulated miRNAs in human TLE-HS tissue (MS Word). (DOC) [file pone.0035921.s004.doc]

**Supplementary data Figure S4**


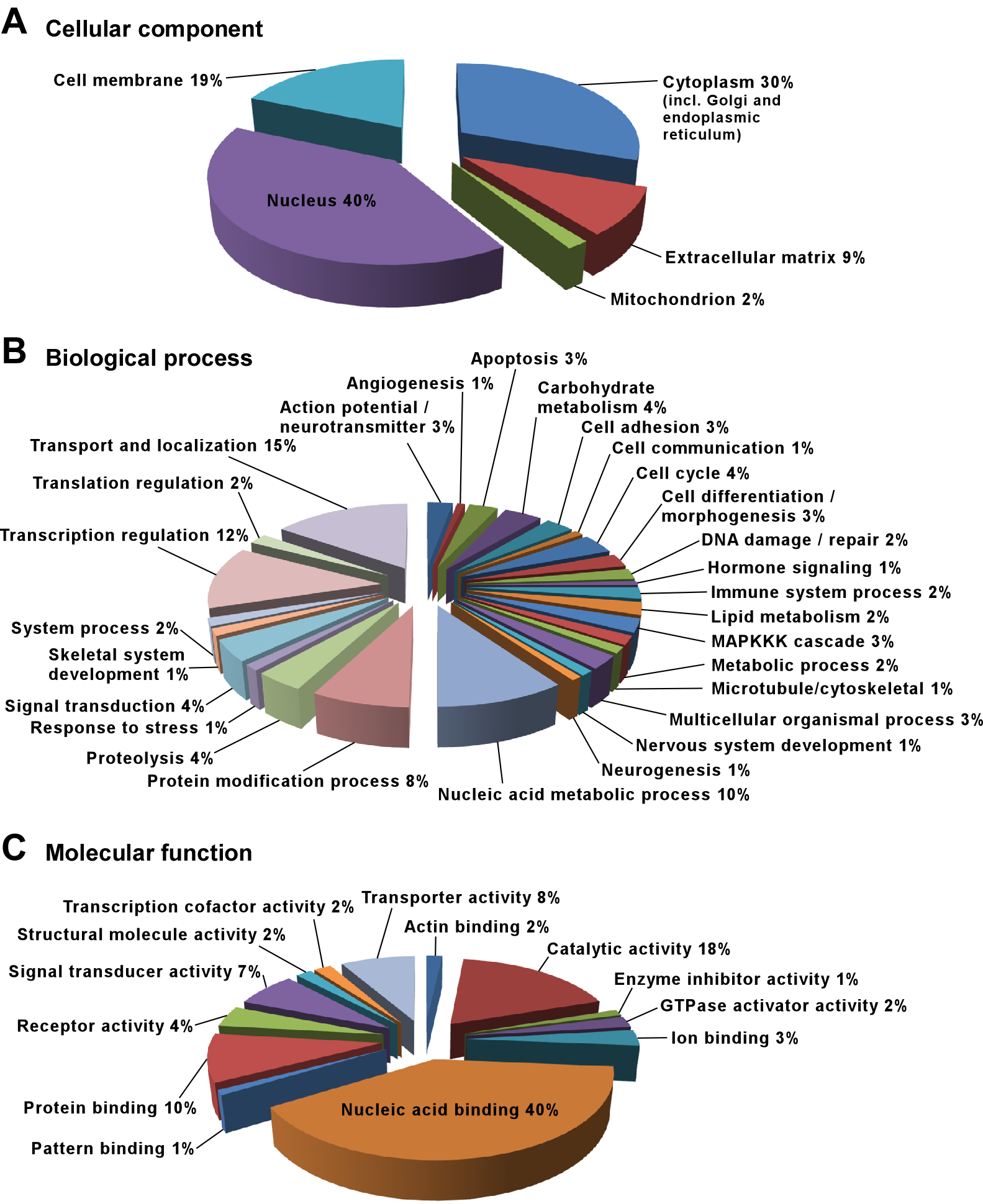


**Supplementary data Figure S4.** *Bioinformatic analysis of genes impacted by significantly down-regulated miRNAs in human TLE-HS.* (A) Pie chart depicting the cellular component for the genes predicted to be targeted by the down-regulated miRNAs in human TLE-HS tissue. (B) Pie chart depicting the biological processes of the genes predicted to be regulated by the miRNAs. (C) Pie chart depicting the molecular functions of the genes predicted to be affected by the reduced miRNAs. *Key*; MAPKKK, mitogen-activated protein kinase kinase kinase.
