# Supplementary material for: Reduced Mature MicroRNA Levels in Association with Dicer Loss in Human Temporal Lobe Epilepsy with Hippocampal Sclerosis
Source: PLoS One. 2012 May 15;7(5):e35921. doi: 10.1371/journal.pone.0035921 (PMC3352899; doi:10.1371/journal.pone.0035921)
Supplement: Table S1 — Top 40 miRNAs expressed in autopsy control human hippocampus. Table listing the 40 mature miRNAs in autopsy control human hippocampus most enriched based on Ct value (MS Word). (DOC) [file pone.0035921.s005.doc]

**Supplementary Table S1.** Top 40 miRNAs expressed in autopsy control human hippocampus

| **miRNA** | **CT value** |  |  | **miRNA** | **CT value** |
| --- | --- | --- | --- | --- | --- |
| Hsa-miR-26a | 20.54 |  |  | Hsa-miR-26b | 24.05 |
| Hsa-miR-9-5p | 20.83 |  |  | Hsa-miR-218-5p | 24.08 |
| Hsa-miR-125b-5p | 21.83 |  |  | Hsa-miR-191 | 24.13 |
| Hsa-miR-125a | 21.98 |  |  | Hsa-miR-223 | 24.23 |
| Hsa-miR-222 | 22.24 |  |  | Hsa-miR-30a-5p | 24.23 |
| Hsa-miR-16-5p | 22.43 |  |  | Hsa-miR-433 | 24.28 |
| Hsa-miR-30c-5p | 22.57 |  |  | Hsa-miR-221-3p | 24.49 |
| Hsa-miR-127 | 22.65 |  |  | Hsa-miR-328 | 24.51 |
| Hsa-miR-132-3p | 22.90 |  |  | Hsa-miR-19b-3p | 24.56 |
| Hsa-miR-24-3p | 23.22 |  |  | Hsa-miR-let-7c | 24.56 |
| Hsa-miR-204-5p | 23.36 |  |  | Hsa-miR-29a | 24.59 |
| Hsa-miR-137 | 23.38 |  |  | Hsa-miR-7 | 24.68 |
| Hsa-miR-126 | 23.71 |  |  | Hsa-miR-9* | 24.88 |
| Hsa-miR-149 | 23.73 |  |  | Hsa-miR-181b | 24.95 |
| Hsa-miR-30b-5p | 23.74 |  |  | Hsa-miR-484 | 25.02 |
| Hsa-miR-let 7b-5p | 23.74 |  |  | Hsa-miR-103a-3p | 25.03 |
| Hsa-miR-100-5p | 23.75 |  |  | Hsa-miR-20a-5p | 25.13 |
| Hsa-miR-92 | 23.93 |  |  | Hsa-miR-99a-5p | 25.13 |
| Hsa-miR-342 | 23.96 |  |  | Hsa-miR-let-7g | 25.15 |
| Hsa-miR-331-3p | 24.02 |  |  | Hsa-miR-140 | 25.18 |
|  |  |  |  |  |  |

Relative enrichment based on Ct value. *from the opposite arm of the miR-9 precursor
